# Cruciferous Vegetable Intake and Bulky DNA Damage within Non-Smokers and Former Smokers in the Gen-Air Study (EPIC Cohort)

**DOI:** 10.3390/nu14122477

**Published:** 2022-06-15

**Authors:** Marco Peluso, Armelle Munnia, Valentina Russo, Andrea Galli, Valeria Pala, Yvonne T. van der Schouw, Matthias B. Schulze, Elisabete Weiderpass, Rosario Tumino, Calogero Saieva, Amiano Exezarreta Pilar, Dagfinn Aune, Alicia K. Heath, Elom Aglago, Antonio Agudo, Salvatore Panico, Kristina Elin Nielsen Petersen, Anne Tjønneland, Lluís Cirera, Miguel Rodriguez-Barranco, Verena Katzke, Rudolf Kaaks, Fulvio Ricceri, Lorenzo Milani, Paolo Vineis, Carlotta Sacerdote

**Affiliations:** 1 Research Branch, Regional Cancer Prevention Laboratory, ISPRO-Study, Prevention and Oncology Network Institute, 50139 Florence, Italy; a.munnia@ispro.toscana.it (A.M.); v.russo@ispro.toscana.it (V.R.); 2 Department of Experimental and Clinical Biomedical Sciences, University of Florence, 50139 Florence, Italy; a.galli@dfc.unifi.it; 3 Epidemiology and Prevention Unit, Fondazione IRCCS Istituto Nazionale dei Tumori di Milano, 20133 Milan, Italy; valeria.pala@istitutotumori.mi.it; 4 Julius Center for Health Sciences and Primary Care, University Medical Center Utrecht, Utrecht University, 3508 GA Utrecht, The Netherlands; yschouw@umcutrecht.nl; 5 Department of Molecular Epidemiology, German Institute of Human Nutrition Potsdam-Rehbruecke, 14558 Nuthetal, Germany; mschulze@dife.de; 6 Institute of Nutritional Science, University of Potsdam, 14558 Nuthetal, Germany; 7 International Agency for Research on Cancer, World Health Organization, 69372 Lyon, France; weiderpasse@iarc.fr; 8 Hyblean Association for Epidemiological Research, AIRE ONLUS, 97100 Ragusa, Italy; rtuminomail@gmail.com; 9 Cancer Risk Factors and Life-Style Epidemiology Unit, ISPRO-Study, Prevention and Oncology Network Institute, 50139 Florence, Italy; c.saieva@ispro.toscana.it; 10 Ministry of Health of the Basque Government, Sub Directorate for Public Health and Addictions of Gipuzkoa, 20014 San Sebastian, Spain; epicss-san@euskadi.eus; 11 Biodonostia Health Research Institute, Epidemiology of Chronic and Communicable Diseases Group, 20014 San Sebastián, Spain; 12 Spanish Consortium for Research on Epidemiology and Public Health (CIBERESP), Instituto de Salud Carlos III, 28029 Madrid, Spain; 13 Department of Epidemiology and Biostatistics, School of Public Health, Imperial College London, London W2 1PG, UK; d.aune@imperial.ac.uk (D.A.); a.heath@imperial.ac.uk (A.K.H.); k.aglago@imperial.ac.uk (E.A.); 14 Unit of Nutrition and Cancer, Catalan Institute of Oncology-ICO, 08908 L’Hospitalet de Llobregat, Spain; a.agudo@iconcologia.net; 15 Nutrition and Cancer Group, Epidemiology, Public Health, Cancer Prevention and Palliative Care Program, Bellvitge Biomedical Research Institute-IDIBELL, 08908 L’Hospitalet de Llobregat, Spain; 16 Department of Clinical Medicine and Surgery, Federico II University, 80138 Naples, Italy; spanico@unina.it; 17 Danish Cancer Society Research Center, Diet, Cancer and Health, DK-2100 Copenhagen, Denmark; kripet@cancer.dk (K.E.N.P.); annet@cancer.dk (A.T.); 18 Department of Public Health, University of Copenhagen, DK-2100 Copenhagen, Denmark; 19 Department of Epidemiology, Regional Health Council-IMIB–Arrixaca, 30120 Murcia, Spain; lluis.cirera@carm.es; 20 CIBER de Epidemiología y Salud Pública (CIBERESP), 28028 Madrid, Spain; miguel.rodriguez.barranco.easp@juntadeandalucia.es; 21 Department of Social and Health Sciences, Murcia University, 30100 Murcia, Spain; 22 Escuela Andaluza de Salud Pública (EASP), 18011 Granada, Spain; 23 Instituto de Investigación Biosanitaria ibs.GRANADA, 18012 Granada, Spain; 24 Division of Cancer Epidemiology, German Cancer Research Center (DKFZ), 69120 Heidelberg, Germany; v.katzke@dkfz-heidelberg.de (V.K.); r.kaaks@dkfz-heidelberg.de (R.K.); 25 Department of Clinical and Biological Sciences, University of Turin, 10124 Turin, Italy; fulvio.ricceri@unito.it (F.R.); lorenzo.milani@edu.unito.it (L.M.); 26 Unit of Cancer Epidemiology, Città Della Salute e Della Scienza University-Hospital and Center for Cancer Prevention (CPO), 10126 Turin, Italy; carlotta.sacerdote@cpo.it; 27 MRC Centre for Environment and Health School of Public Health, Imperial College LondonSt Mary’s Campus, Norfolk Place, London W2 1PG, UK; p.vineis@imperial.ac.uk

**Keywords:** EPIC, diet, cruciferous vegetables, DNA damage, B(a)P-adducts, bulky oxidative lesions

## Abstract

Epidemiologic studies have indicated that cruciferous vegetables can influence the cancer risk; therefore, we examined with a cross-sectional approach the correlation between the frequent consumption of the total cruciferous vegetables and the formation of bulky DNA damage, a biomarker of carcinogen exposure and cancer risk, in the Gen-Air study within the European Prospective Investigation into Cancer and Nutrition (EPIC) cohort. DNA damage measurements were performed in the peripheral blood of 696 of those apparently healthy without cancer controls, including 379 never-smokers and 317 former smokers from seven European countries by the ^32^P-postlabeling assay. In the Gen-Air controls, the median intake of cruciferous vegetables was 6.16 (IQR 1.16–13.66) g/day, ranging from 0.37 (IQR 0–6.00) g/day in Spain to 11.34 (IQR 6.02–16.07) g/day in the UK. Based on this information, participants were grouped into: (a) high consumers (>20 g/day), (b) medium consumers (3–20 g/day) and (c) low consumers (<3.0 g/day). Overall, low cruciferous vegetable intake was correlated with a greater frequency of bulky DNA lesions, including benzo(a)pyrene, lactone and quinone-adducts and bulky oxidative lesions, in the adjusted models. Conversely, a high versus low intake of cruciferous vegetables was associated with a reduction in DNA damage (up to a 23% change, *p* = 0.032); this was particularly evident in former smokers (up to a 40% change, *p* = 0.008). The Generalized Linear Regression models indicated an overall Mean Ratio between the high and the low consumers of 0.78 (95% confidence interval, 0.64–0.97). The current study suggests that a higher intake of cruciferous vegetables is associated with a lower level of bulky DNA adducts and supports the potential for cancer prevention strategies through dietary habit changes aimed at increasing the consumption of cruciferous vegetables.

## 1. Introduction

Diet has an important role in health and disease and is considered to be an important modifiable environmental factor able to influence the cancer risk [1]. Diets rich in fruits and vegetables have been associated with a lower risk of colorectal cancer [1]. The evidence from several epidemiology studies has suggested that the frequent intake of cruciferous vegetables is linked with a reduced cardiovascular mortality, incidence of renal cell carcinoma, breast and lung cancer mortality and colorectal cancer risk in women [2,3]. A meta-analysis demonstrated an inverse association between the intake of cruciferous vegetables and lung cancer risk with an odds ratio (OR) of 0.81 (95% confidence interval (CI), 0.74–0.89) between the highest and the lowest intake categories [4]. Our diet can influence our susceptibility to carcinogens. Studies have demonstrated that the frequent consumption of fruits and vegetables that are rich in antioxidants and other micronutrients has a protective effect against the generation of DNA damage [5]. A randomized controlled trial examining the association between antioxidant vitamin supplementation and DNA damage has found a significant protective effect but only among women, and a cross-sectional investigation showed an inverse association of DNA damage with the plasma levels of certain micronutrients, such as retinol, α-tocopherol and γ-tocopherol [5]. Moreover, certain kinds of foods contain bioactive compounds with antioxidant and anti-inflammatory properties that can be used to develop prevention programs against chronic diseases, such as cardiovascular disease, neurodegenerative disease and gastrointestinal cancer [6,7].

Conversely, epidemiologic reports have associated diets characterized by the frequent consumption of red and processed meats with an increased risk of colorectal cancer [1]. This association is related to the consumption of food mutagens and influenced by the extent to which these chemicals are activated by metabolic enzymes, mainly via the P450 cytochrome pathway [8]. Meats when cooked at high temperatures become oxidized, generating heterocyclic amines and free radicals [9]. Other mutagens, such as polycyclic aromatic hydrocarbons (PAHs), are present in grilled and barbecued meat and processed foods or occur in the food chain due to environmental pollution [8]. PAHs are a recognized class of carcinogens with well-established genotoxic properties [5]. PAHs induce cytochrome P450 monooxygenases that are the key metabolizing enzymes in the metabolic activation of carcinogens such as diol-epoxides, radical cations and o-quinones able to directly react with the DNA by forming DNA adducts [10]. Free radicals, such as reactive oxygen species, are also able to induce bulky oxidative DNA adducts by direct DNA oxidation [11]. The principal biological consequences of DNA damage formation is the decline in the physiological mechanisms designed to maintain cell repair and keep up the metabolic homeostasis, leading to tissue injury and cell transformation [12]. DNA damage, unless fully repaired, can lead to mutations, including in oncogenes and tumor suppressors, initiating the process of carcinogenesis [5]. Moreover, we provide evidence that the generation of DNA damage at a single nucleotide resolution causes characteristic signatures at the site of mutations along the *TP53* sequence, indicating a causal relationship between DNA damage and cancer [13,14].

Early on, we investigated the prospective ability of DNA damage to predict cancer among the former smokers and non-smokers in Gen-Air, a case–control study nested in the European Prospective Investigation into Cancer and Nutrition (EPIC) [15]. The principal advantage of the Gen-Air study was that the level of DNA damage was detected in blood samples collected several years before the onset of cancer (median follow-up of 7 years); therefore, the adducts were not influenced by the early effects of the cancer disease itself. In that investigation, the adducts were linked to the lung cancer risk, with an OR of 1.86 (95% CI, 0.88–3.93), when comparing detectable versus nondetectable adducts. The association with lung cancer was greater in non-smokers (OR, 4.04 (95% CI 1.06–15.42)) and among the younger age groups. After excluding of the cancers occurring in the first 36 months of the follow-up, the OR was 4.16 (95% CI 1.24–13.88).

In the current study, we decided to evaluate the relationship between the frequent consumption of cruciferous vegetables and DNA damage among 696 non-smoker and former smokers apparently healthy without cancer controls in the Gen-Air cohort within the EPIC study [15]. The formation of DNA damage in peripheral blood leukocytes was analyzed blindly by using the ^32^P-postlabeling technique [16]. Our aim was to investigate the potential of cruciferous vegetables in lowering the formation of DNA damage among a large European healthy population to identify a potential predominant dietary factor to be used in dietary intervention strategies.

## 2. Material and Methods

### 2.1. Selection of Subjects and Collection of Specimens

EPIC is a multicenter European study, coordinated by the IARC, Lyon, in which more than 500,000 healthy volunteers were recruited from twenty-three centers in ten countries, including France, Denmark, Germany, Greece, Italy, the Netherlands, Norway, Spain, Sweden and the United Kingdom. The cohort included both men and women, aged especially in the range of 35–74 years. Detailed dietary and lifestyle histories were obtained mainly through self-administered questionnaires; plus, a 24-h dietary recall through a person-to person interview (in a 10% sample), anthropometric measurements and 30–40 mL peripheral blood specimens were collected. Signed informed consent forms were collected from all participants (except a subgroup of the Oxford cohort who gave consent in postal questionnaires). Gen-Air was a case–control study nested within the EPIC cohort. Its aim was to study the relationship between various types of cancer and air pollution or environmental tobacco smoke. All cancers were diagnosed after recruitment. Only never-smokers or ex-smokers who had not smoked for more than 10 years before enrollment were included. This cutoff point was set to control for the potential residual confounding effect of smoking in ex-smokers. Three controls were matched per case. The matching criteria were sex, age ± 5.0 (years), smoking status (never and former), country of recruitment and follow-up time. The number of participants who met the protocol criteria was 3980 (1046 cases and 2934 controls). The mean follow-up time for those participants was 89 months, with a minimum of 51 months and a maximum at 123 months [15]. The study was approved by the Ethical Committee of the IARC and the local ethical committees of the twenty-three recruitment centers in Europe (*n* = 13/94). We identified 2934 controls who met the protocol criteria. Of these subjects, 1149 had peripheral blood samples. Blood samples of the controls for the centers that gave ethical approval were sent to the laboratories for investigation: controls from France, Denmark, Germany, Italy, the Netherlands, Spain and United Kingdom were considered for the analysis. In the current cross-sectional investigation, the study population consisted of 696 apparently healthy without cancer controls, including 379 never-smokers and 317 former smokers from seven European countries.

### 2.2. Dietary and Lifestyle Variables

At enrollment, the weight, height and waist and hip circumferences were measured for each participant. Detailed information was collected on their reproductive history, physical activity, tobacco smoking, medical and occupation histories, education levels and other socioeconomic variables. Dietary information on the frequency of consumption of more than 120 foods and drinks was obtained by dietary questionnaires developed and validated in a pilot phase in each participating country.

### 2.3. DNA Adduct Analysis

DNA was extracted using a method that requires RNase and proteinase treatments and extraction with organic solvents, with the exception of the Danish samples, which were extracted and purified by a salting-out procedure [15]. Coded DNA was stored at −80 °C until the laboratory analysis. The frequency of DNA damage was investigated blindly by ^32^P-postlabeling [15] using a chromatographic system efficient detection of different kinds of DNA damage, including benzo(a)pyrene, lactone and quinone-adducts and bulky oxidative lesions [17,18,19]. The detection and quantification of DNA adducts were performed by storage phosphor imaging techniques employing intensifying screens from Molecular Dynamics (Sunnyvale, CA, USA). The intensifying screens were scanned using a Typhoon 9210 (Amersham, Pleasanton, CA, USA). The software used to process the data was ImageQuant (version 5.0) from Molecular Dynamics, Pleasanton, CA, USA. DNA adducts were expressed as relative adduct labeling (RAL) = pixels in adducted nucleotides/pixels in normal nucleotides. The mean adduct level was corrected across experiments based on the recovery of the carcinogen-adducted DNA standards: one prepared in vitro by the reaction of B(a)P diolepoxide (BPDE, ^3^H-labeled) with DNA according to a published method [20], while the other consisted of a DNA sample extracted i.p. from the hepatic tissue of four mice treated with 1.0 mg of [7,8-^3^H]B(a)P for 24 h [21]. The level of B(a)P adducts in the livers of the B(a)P-treated experimental animals was also quantified as B(a)P-tetrols released from the hydrolysis of macromolecules and measured by GC-MS [21].

### 2.4. Statistical Analysis

The association between cruciferous vegetable intake and DNA damage was examined overall, as well as after stratification, by the smoking status, considering former and never-smokers. The study population was divided into three dietary categories of cruciferous vegetable intake, as follows: (a) high consumers (>20 g/day), (b) medium consumers (3–20 g/day) and (c) low consumers (<3.0 g/day). We used adducts as a continuous variable after log transformation. The mean adduct level across the dietary categories of cruciferous vegetables was compared by an analysis of covariance, introducing into each model terms for the age (years); sex; country; smoking status; blood sampling time (winter, midseason and summer); body mass index (BMI, kg/m^2^); alcohol consumption (g/day) and daily energy intake (kcal/day). The variable selection was based on associations with the adduct level found in previous investigations [5,22]. We used multivariate statistical analyses using a Generalized Linear Regression model with log–link in order to evaluate the association between cruciferous vegetable consumption and DNA damage, introducing terms for age, sex, country, smoking status, blood sampling season, BMI, alcohol consumption and daily energy intake as further adjustment variables [23]. The results were interpreted as the Mean Ratio (MR) value between the means of the DNA adducts and categorical cruciferous vegetable intake. The confidence level assumed for statistical significance was 95%. Data were analyzed using SAS9.3 (StataCorp LLC, College Station, TX, USA) and SPSS 20.0 (IBM SPSS Statistics, New York, NY, USA).

## 3. Results

### 3.1. Cruciferous Vegetables

Table 1 and Table 2 show the participant characteristics and mean and median intakes of cruciferous vegetables. No difference in the median dietary consumption of cruciferous vegetables was found between men and women or between never-smokers and former smokers, and there seemed not to be differences between the different blood sampling seasons. The intake of cruciferous vegetables was highest in the UK (median 11.34 g/day) and lowest in Spain (0.37 g/day). Older people tended to have a diet richer in cruciferous vegetables than younger people (Table 2). No differences were observed in the mean and median levels of DNA damage by recruitment after correction for confounding factors (Table 2).

### 3.2. Cruciferous Vegetable Intake and DNA Damage

A characteristic BPDE-DNA chromatographic pattern was detected in the chromatogram of the BPDE adduct standard. The visual investigation showed a major spot, due to the N^2^ guanine substitution by anti-b(*a*)p 7,8-dihydrodiol 9,10-oxide (anti-BPDE). The level of BPDE-DNA adduct was 1030 ± 670 (Standard Deviation, SD) in BPDE-treated calf-thymus DNA by ^32^P-postlabeling. Conversely, the level of B(a)P-related DNA adducts in the livers of the experimental animals was 190 ± 90 (SD) adducts per 10^9^ normal nucleotides, thereby at level comparable to the 205 adducts per 10^9^ anormal nucleotides detected in the same hepatic tissues by the GC-MS technique [21].

Table 3 shows the adjusted level of bulky DNA adduct values, expressed as RAL per 10^9^ normal nucleotides, among the three dietary cruciferous vegetable intake categories in the Gen-Air cohort obtained from the analysis of covariance. The frequency of the DNA damage was inversely associated with cruciferous vegetable consumption. Reduced adduct frequencies were generally found in the high consumers of cruciferous vegetables as compared to the low consumers. The bulky adduct frequency was 23% lower in participants who reported a higher intake of cruciferous vegetables as compared to the low consumers (*p* = 0.025). After stratification by smoking status, a not statistically significant change was observed in the never-smokers, whereas a significant reduction was detected in former smokers in both the medium- and high-intake categories compared to low-intake. For the medium-intake category, the reduction was 12% (*p*-value = 0.036), while the highest category had a 40% lower adduct level compared with the lowest category (*p*-value = 0.016).

The overall Mean Ratio (MR) between the high and the low categories of cruciferous vegetables consumption was 0.79 (95% CI 0.64–0.97, *p* = 0.026) after an adjustment for the confounding factors (Table 4). A reduction in the adduct level was also found between the medium and the low consumers of cruciferous vegetables, with a MR of 0.84 (95% CI 0.73–0.98, *p* = 0.030). After stratification by the smoking status, a MR of 0.65 (95% CI 0.48–0.88, *p* = 0.006) was observed between the high and the low consumers of cruciferous vegetables among former smokers. Additionally, former smokers with medium compared to low intakes had a MR of 0.79 (95% CI 0.63–0.99, *p* = 0.043). Among never-smokers, instead, the estimates were not significant for either the high or medium levels of cruciferous vegetable intake compared to the lowest.

## 4. Discussion

One of the main advantages of molecular epidemiology is that surrogate biomarkers can be used in identifying foods and specific nutrients that seem to be linked with a reduced cancer risk [24]. Thus, exploring the association between dietary habits and the levels of biomarkers related to carcinogen exposure and cancer risk is highly relevant in public health. Early on, we examined the associations between dietary habits with DNA and protein damage in leucocytes and erythrocytes of non-smokers and former smokers from Gen-Air to the EPIC cohort [25]. In that study, inverse associations of bulky DNA adducts with a dietary fiber intake of vitamin E were demonstrated, while there were inverse relationships between 4-aminobiphenyl-Hb adducts and fiber and fruit intakes. In the present study, we investigated by a cross-sectional approach the association between the dietary consumption of cruciferous vegetables and the frequency of bulky DNA adducts, a biomarker of carcinogen exposure and cancer risk [5,26] among the same Gen-Air cohort [15]. Overall, we found that a lower cruciferous vegetable intake was correlated with a higher level of different types of bulky DNA adducts, such as benzo(a)pyrene, lactone and quinone adducts and bulky oxidative lesions [17,18,19], in the adjusted models. Conversely, individuals with a frequent intake of cruciferous vegetables showed a reduction of bulky DNA lesions as compared to lower consumers. The findings can reflect the overall antigenotoxic effects due to high intakes of cruciferous vegetables in the amounts and varieties prepared by and consumed by the EPIC volunteers. A multivariate regression analysis demonstrated the presence of a negative linear correlation between the adducts and intakes of cruciferous vegetables in the Gen-Air cohort.

Our study is in agreement with different studies, such as a cross-sectional study within the EPIC Italy cohort [27] and a dietary intervention trial [28], where about a 20% reduction of the DNA damage was observed in frequent consumers of cruciferous vegetables as compared to low consumers. Over the last years, several studies have been conducted to evaluate the potential antigenotoxic effects of various dietary patterns or specific foods by measuring the frequency of bulky DNA adducts [5]. Large cohorts showed that the frequency of DNA damage was inversely correlated with the consumption of fruits and vegetables [5]. The traditional Thai diet rich in flavor and condiments with anticarcinogenic properties [29] was found to be inversely associated with the formation of lipid peroxidation-related DNA adducts, e.g., the 3-(2-deoxy-β-D-erythro-pentafuranosyl)pyrimido (1,2-α)purin-10(3H)-one deoxyguanosine adduct in the Map Tha Phut cohort [30]. The Mediterranean diet, a diet characterized by the frequent consumption of ascorbic acid, beta-carotene, alpha-tocopherol and fatty acids, mostly derived from olive and other vegetable oils, was found to be highly protective against the production of DNA damage [27]. A protective antigenotoxic food pattern, rich in fresh fruits, raw or cooked vegetables and with a low saturated fat intake, was identified in the EPIC Italy cohort [27,31,32]. An inverse association of DNA damage with vegetable intake was found in human colorectal mucosa within the EPIC cohort in the United Kingdom [33]. The maternal consumption of fruits and vegetables during pregnancy was found to be inversely associated with the frequency of DNA damage in the cord blood of newborns in the NewGeneris study [34]. The antigenotoxic properties of cruciferous vegetables have still been demonstrated in peripheral blood human lymphocytes [35], where an inverse correlation with the cruciferous vegetable intake was observed. Moreover, purified phytochemicals from cruciferous vegetables have been found to reduce the generation of colonic DNA adducts formed by an aromatic amine, 2-amino-1-methyl-6-phenylimidazo[4,5-b]pyridine, in experimental animals [36,37]. Indole-3-carbinol, contained in cabbage, cauliflower, broccoli and brussels sprouts, has been associated with adduct production inhibition [38].

In the current study, the anticancer effect on bulky DNA adducts was specifically cruciferous as opposed to all other foodstuffs, although people with a high intake of cruciferous are likely to also show a high intake of other vegetables and fruits rich in similar bioactive phytochemicals. However, when the relationship between the levels of bulky DNA adducts and other foodstuffs, such as fibers, fruit, legumes, vegetables, and, also, estimates of the intake of vitamins C and E and β-carotene, was previously examined in the Gen-Air cohort, there were no appreciable differences in any dietary variable except for fibers [23]. On the other hand, we have not analyzed the plasmatic levels of cruciferous bioactive compounds with antioxidant properties in plasma; therefore, we cannot clearly demonstrate that the effects of a cruciferous intake are really due to their antioxidant properties. Thereby, the hypothesis that a higher intake of cruciferous containing bioactive antioxidants is associated with a reduced leukocyte level of bulky adducts needs to be addressed with additional studies. Furthermore, from a public health point of view, the message that to eat vegetables decreases the risk of developing cancers is also strengthened if the reduction of bulky DNA adducts is due to the synergic effect of the intake of cruciferous with other vegetables.

Cruciferous or *Brassicaceae* vegetables, including broccoli, cabbage, cauliflower and brussels sprouts, are high in micronutrients and fiber and contain vitamins C and E, antioxidant enzymes, polyphenols and sulfur–organic compounds [39,40]. The beneficial effects of cruciferous vegetables on human health are thought to be largely mediated by different plant components, such as isothiocyanates and indoles [36,37], that are formed by the hydrolytic action of plant myrosinase or caused by the glucosidases present in the gut microbiota [35]. Other relevant phytochemicals include sulforaphane, which can inhibit the activation of carcinogens, enhance their excretion and induce cell cycle arrest and apoptosis [41]. Bioactive compounds can act by various manners, including by inhibiting cytochrome P450 and inducing glutathione *S*-transferases, quinone reductase and nuclear factor erythroid 2-related factor 2, a transcription factor that has a main role in protecting cells from oxidative injury [42]. Vitamins C and E, antioxidant enzymes and polyphenols can also influence both the pathways of the metabolic activation of carcinogens and repair of DNA adducts [43]. Cruciferous vegetables are an important source of fiber, a heterogeneous group of plant compounds, especially indigestible carbohydrates, including starches, natural compounds that escape digestion in the small intestine. Fiber can reduce DNA adduct formation by diluting food carcinogens in the gastrointestinal tract and by speeding up their transit through the colon [23]. In a clinical trial [44], the intake of butyrylated starch was linked to low DNA damage. Laky et al. [45] reported that extracts of brussels sprouts can decrease the genotoxic effect of carcinogens in experimental cells.

Pesticides are used to control pests and improve agricultural production, and the standards of pesticides used in each country are different. Despite their selectivity of action, a number of agrochemicals have also been reported to be genotoxic using the present assay [46,47,48,49]. The genotoxicity of pesticides is indeed an issue of worldwide concern [50]. For instance, greenhouse floriculturists and open field farms have been associated with a significantly higher level of bulky DNA adduct with respect to nonexposed controls [51,52,53]. Thereby, the effects of pesticide exposure, if any in the Gen-Air cohort, should be compared against the protective effects of cruciferous bioactive compounds on DNA adduct formation rather than being a bias effect. Moreover, all the multivariate analyses were adjusted by country.

In our study, the background frequency of bulky DNA adducts measured in the Gen-Air cohort was of about 7.0 adducts per 10^9^ normal nucleotides, possibly caused by exposure to different environmental carcinogens [15]. In Western countries, the major sources of environmental pollutants are tobacco cigarette smoke, air pollution and diet [54]. In former smokers, DNA damage can also be a consequence of the slow clearance of carcinogens contained in tar and particulates in the respiratory tract of smokers that leads to the continuous activation of tobacco smoke-related carcinogens [55]. Some adducts can even persist, because certain damage is repaired slowly or entirely resistant to the repair mechanisms [56]. The formation of DNA adducts is generally thought to be an event that occurs in the early, initiating stages of carcinogenesis and is considered to be “necessary but not sufficient”; however, a meta-analysis of 22 adduct studies with bronchial adducts, for a total of 1091 subjects, 887 lung cancer cases and 204 healthy individuals, demonstrated a significant difference, with lung cancer patients having a 103% greater amount of bronchial DNA damage than the controls [26]. In that study, the bronchial adducts were not simply related to carcinogen exposure but also a cause of chemical-induced lung cancer.

The risk assessment of chemical carcinogens is one the major aims in public health, since low levels of carcinogenic substances in foods and the environment are always present and often not completely avoidable [57]. Otteneder and Lutz [58] examined the quantitative relationship between DNA adducts and tumor incidences in livers for 27 major carcinogens in 2-year bioassays, demonstrating that the measurement of DNA damage in the target tissue could be considered as an individual cancer risk marker. For the cancer risk assessment, the extrapolation of the relationship between the dose and tumor incidence from 2-year bioassays in humans is difficult; nevertheless, the significant reduction of the DNA damage, particularly evident in former smokers (40% change), could be theoretically sufficient to detect a lower level of at least genetic mutations, which, of course, has to be verified experimentally. From this perspective, our results suggested a potential modulation of carcinogen metabolism by using natural plant components of the cruciferous vegetable group in a large European healthy population within the EPIC cohort.

A main strength of this study is that the correlation between cruciferous vegetables and DNA adducts was analyzed in a sufficiently powered dataset, whereas a limitation was the relatively low mean levels of daily consumption of cruciferous vegetables in the Gen-Air cohort (10.3 ± 0.3 g/day) relative to the other countries [59], such as 23.0 g/day in the United States, 60.0 g/day in Japan and 121.0 g/day in Hong Kong. Additionally, the adherence to a diet was not explicitly investigated, and the UK cohort consisted of vegetarians alone. In addition, we cannot rule out that other unidentified factors could be associated with the lower generation of bulky DNA adducts in the high consumers of cruciferous vegetables. Some of the protective effects could also be due to interactions and synergisms between several dietary components.

## 5. Conclusions

The diet is usually a complex of both nutritive and non-nutritive food constituents; thus, the search for specific factors with health protective effects among a dietary pattern is generally stronger than the evidence based on individual foods. Nevertheless, the findings from this large prospective European study provide additional evidence for a beneficial effects of a diet rich in cruciferous vegetables with respect to their antigenotoxic properties, especially among former smokers. Our study supports the substantial potential for cancer prevention strategies based on the frequent consumption of these vegetables to act against DNA damage generation and, possibly, cancer development.

## Figures and Tables

**Table 1:** Demographic characteristics of the Gen-Air cohort.

Variable	
Age, years (Median, IQR)	61.03 (55.18–65.43)
Sex (N, %)	
Male	360 (51.79)
Female	336 (48.21)
Smoking status (N, %)	
Never-smokers	379 (54.45)
Former smokers	317 (45.55)
Country (N, %)	
France	12 (1.72)
Italy	105 (15.09)
Spain	91 (13.07)
UK	234 (33.62)
Netherlands	53 (7.61)
Germany	140 (20.11)
Denmark	61 (8.76)
Blood sampling season (N, %)	
Winter	143 (20.52)
Mid-season	189 (27.12)
Summer	357 (51.22)
Missing	7 (1.14)
Energy kcal/day (Median, IQR)	2081 (1673–2535)
Cruciferous vegetables g/d (Median, IQR)	6.16 (2.16–13.66)
Alcohol g/day (Median, IQR)	5.86 (0.61–15.13)

IQR, interquartile range.

**Table 2:** Mean daily intake of cruciferous vegetables and mean level of DNA damage by subject demographic characteristics.

Variable	N	Mean ± SD Intake of Cruciferous Vegetables (g/day)	Median (IQR) Intake of Cruciferous Vegetables (g/day)	*p*	Mean ± SD Level of DNA Damage (RAL)	Median (IQR) Level of DNA Damage (RAL)	*p*
Recruitment age							
<55 years	171	11.18 ± 16.66	5.49 (1.84–14.28)	0.01	0.68 ± 0.64	0.60 (0.20–0.90)	0.53
55–65 years	367	9.78 ± 12.86	6.02(2.00–12.20)		0.69 ± 0.54	0.60 (0.30–1.00)	
>65 years	158	11.98 ± 12.05	7.47 (4.9–16.66)		0.70 ± 0.55	0.60 (0.30–1.00)	
Sex							
Male	361	10.47 ± 11.21	6.30 (2.56–14.00)	0.13	0.69 ± 0.57	0.60 (0.30–0.95)	0.96
Female	336	10.80 ± 16.01	6.02 (1.96–12.76)		0.69 ± 0.57	0.59 (0.20–1.00)	
Smoking status							
Never-smokers	379	10.66 ± 14.98	6.02 (1.96–13.71)	0.34	0.70 ± 0.58	0.60 (0.20–1.00)	0.98
Former smokers	317	10.59 ± 12.11	6.27 (2.56–13.48)		0.69 ± 0.56	0.54 (0.30–1.00)	
Country							
France	12	21.06 ± 24.01	8.25 (4.63–38.85)	<0.01	0.73 ± 0.34	0.75 (0.50–1.05)	0.14
Italy	105	13.36 ± 12.58	9.49 (4.49–17.74)		0.72 ± 0.66	0.50 (0.20–1.00)	
Spain	91	4.59 ± 9.54	0.37 (0.00–6.00)		0.69 ± 0.58	0.60 (0.20–1.10)	
UK	234	14.04 ± 14.46	11.34 (6.02–16.87)		0.72 ± 0.57	0.60 (0.30–1.00)	
Netherlands	53	5.93. ± 9.17	3.78 (2.00–6.40)		0.59 ± 0.63	0.30 (.010–0.80)	
Germany	140	8.93 ± 15.52	4.75 (2.08–9.25)		0.66 ± 0.55	0.60 (0.20–0.80)	
Denmark	61	7.45 ± 7.79	5.39 (2.99–7.93)		0.70 ± 0.40	0.70 (0.40–1.00)	
Blood sampling season							
Winter	143	11.79 ± 16.04	7.03 (1.96–14.28)	0.18	0.74 ± 0.58	0.60 (0.30–1.10)	0.30
Mid-season	189	8.98 ± 1.57	6.02 (1.96–11.87)		0.73 ± 0.65	0.60 (0.30–1.10)	
Summer	357	11.03 ± 14.14	6.26 (2.56–14.00)		0.65 ± 0.51	0.51 (0.20–0.90)	
Missing	7	11.03 ± 16.81	4.79 (0.98–16.66)		0.98 ± 0.49	0.80 (0.54–1.50)	
Energy intake (kcal/day)							
<1812	232	10.05 ± 15.58	5.48 (1.56–12.09)	0.02	0.74 ± 0.60	0.60 (0.27–1.10)	0.30
1812–2373	232	10.59 ± 11.60	6.83 (2.94–13.90)		0.64 ± 0.51	0.50 (0.25–0.85)	
>2374	232	11.24 ± 13.80	6.41 (2.43–14.89)		0.70 ± 0.59	0.60 (0.20–1.00)	

IQR, interquartile range; RAL, relative adduct labeling per 10^9^ normal nucleotides; SD, standard deviation.

**Table 3:** Adjusted mean level of DNA damage by dietary cruciferous vegetable categories and smoking habits.

Intake of Cruciferous Vegetables	N	Adjusted Adduct Level ± SD ^a^	Adduct Change	*p*-Value ^b^	*p*-Value for Trend ^c^
Low intake (<3.0 g/day)	217	0.79 ± 0.05		Reference	
Medium intake (3–20 g/day)	377	0.66 ± 0.04	−16.44%	0.036	
High intake (>20 g/day)	102	0.60 ± 0.06	−23.32%	0.032	0.078
After smoking status stratification					
Never-smokers					
Low intake (<3.0 g/day)	125	0.76 ± 0.06		Reference	
Medium intake (3–20 g/day)	196	0.67 ± 0.05	−11.94%	0.360	
High intake (>20 g/day)	58	0.69 ± 0.09	−9.18%	0.719	0.760
Former smokers					
Low intake (<3.0 g/day)	92	0.84 ± 0.07		Reference	
Medium intake (3–20 g/day)	181	0.66 ± 0.05	−12.17%	0.052	
High intake (>20 g/day)	44	0.51 ± 0.09	−39.87%	0.008	0.020

^a^ From the analysis of covariance, including terms for age (years); sex; country; smoking status; blood sampling time (winter, midseason and summer); body mass index (BMI, kg/m^2^); alcohol consumption (g/day) and daily energy intake (kcal/day). RAL, relative adduct labeling per 10^9^ normal nucleotides; SD, standard deviation. ^b^ From Dunnett’s test for multiple comparisons. ^c^ From the GLM model, including terms for age (years); sex; country; smoking status; blood sampling time (winter, midseason and summer); BMI (kg/m^2^); alcohol consumption (g/day) and daily energy intake (kcal/day).

**Table 4:** Mean Ratio (MR) of DNA adducts and 95% Confidence Interval (CI) by dietary categories and smoking habits.

Intake of Cruciferous Vegetables	MR and 95% CI ^a^	*p* Value ^a^
Low intake (<3.0 g/day)	1.00 (reference)	
Medium intake (3–20 g/day)	0.84 (95% CI 0.73–0.98)	0.030
High intake (>20 g/day)	0.79 (95% CI 0.64–0.97)	0.026
After smoking status stratification		
Never-smokers		
Low intake (<3.0 g/day)	1.00 (reference)	
Medium intake (3–20 g/day)	0.89 (95% CI 0.73–1.09)	0.267
High intake (>20 g/day)	0.92 (95% CI 0.69–1.23)	0.596
Former smokers		
Low intake (<3.0 g/day)	1.00 (reference)	
Medium intake (3–20 g/day)	0.79 (95% CI 0.63–0.99)	0.043
High intake (>20 g/day)	0.65 (95% CI 0.48–0.88)	0.006

^a^ Model adjusted for age (years); sex; country; smoking status; blood sampling time (winter, midseason and summer); BMI (kg/m^2^); alcohol consumption (g/day) and daily energy intake (kcal/day).

## Data Availability

Data are available from IARC upon request and following approval by the EPIC Steering committee.
